# Detection of occult paroxysmal atrial fibrilation by implantable long-term electrocardiographic monitoring in cryptogenic stroke and transient ischemic attack population: a study protocol for prospective matched cohort study

**DOI:** 10.1186/s12872-015-0160-8

**Published:** 2015-12-03

**Authors:** Andrea Petrovičová, Egon Kurča, Miroslav Brozman, Jozef Hasilla, Pavel Vahala, Peter Blaško, Andrea Andrášová, Robert Hatala, Luboš Urban, Štefan Sivák

**Affiliations:** Department of Neurology, Faculty Hospital, Constantine Philosopher University, Špitálska 6, 94901 Nitra, Slovak Republic; Clinic of Neurology, Jessenius Faculty of Medicine, Comenius University, Kollárova 2, 03659 Martin, Slovak Republic; Clinic of Cardiology, Faculty Hospital, Constantine Philosopher University, Špitálska 6, 94901 Nitra, Slovak Republic; Kardiocentrum Nitra s.r.o, Špitálska 1, 94901 Nitra, Slovak Republic; Department of Arrhythmias and Cardiac Pacing, The National Institute of Cardiovascular Diseases, Pod Krásnou hôrkou 1, 83348 Bratislava, Slovak Republic

**Keywords:** Transient ischemic attack, Cryptogenic stroke, Atrial fibrillation, Implantable loop recorder, PITX2, ZFHX3

## Abstract

**Background:**

Cardio-embolic etiology is the most frequently predicted cause of cryptogenic stroke/TIA. Detection of occult paroxysmal atrial fibrillation is crucial for selection of appropriate medication.

**Methods:**

Enrolment of eligible cryptogenic stroke and TIA patients began in 2014 and will continue until 2018. The patients undergo long-term (12 months) ECG monitoring (implantable loop recorder) and testing for PITX2 (chromosome 4q25) and ZFHX3 (chromosome 16q22) gene mutations. There will be an appropriate control group of age- and sex-matched healthy volunteers. To analyse the results descriptive statistics, statistical tests for group differences, and correlation analyses will be used.

**Discussion:**

In our study we are focusing on a possible correlation between detection of atrial fibrillation by an implantable ECG recorder, and PITX2 and/or ZFHX3 gene mutations in cryptogenic stroke/TIA patients. A correlation could lead to implementation of this genomic approach to cryptogenic stroke/TIA diagnostics and management. The results will be published in 2018.

**Trial registration:**

ClinicalTrials.gov: NCT02216370.

## Background

Stroke has consistently been the 2^nd^ leading cause of death in the age group of 60 plus worldwide [1]. Recently, it has become the 4^th^ leading cause of death and the 1^st^ leading cause of serious long-term disability in the USA [2]. Accurate definition of stroke etiology is crucial for the most effective management of the disease.

Cryptogenic stroke (CS), according to the TOAST classification, is defined as an ischemic stroke with undeterminable etiology, despite extensive evaluation [3]. Stroke might be cryptogenic for several reasons: 1) evaluation is not carried out at the time of event; 2) investigation is not sufficiently extensive; or 3) there are other unknown causes. Therefore the proportion of CS depends on the speed and wider-than-routine range of diagnostic evaluation. Epidemiological observational trials using standardized stroke etiological classification report 20–40 %, in some cases up to 50 % incidence of unrevealed causes [4–6].

Cardio-embolic stroke is the most frequently predicted cause of CS in studies (58 %) [7, 8]. The most frequent cause of cerebral cardio-embolism, as well as the most common cardiac arrhythmia, is atrial fibrilation (AF). The incidence of stroke in people with non-valvular AF is estimated to be 5 times higher than in people without AF [9]. It has been suggested that paroxysmal AF (PAF) is more prevalent than chronic AF in stroke and TIA patients, and they both appear to carry very similar stroke risk [10].

Episodes of PAF may reoccur with variable frequency and their detection has been made possible by ECG monitoring. Short and usually asymptomatic presentations of PAF (also occult PAF) may remain undetected by routine arrhythmia screening methods. Incidence of newly identified PAF varies widely depending on the type of cardiac monitoring devices, the delay in monitoring after stroke onset and the monitoring duration. Detection rate of PAF with external ECG monitoring in patients after CS has been recorded between 5 and 20 % [11–13]. An insertable cardiac monitor (ICM) automatically provides non-stop long-term ECG recording with very high sensitivity and specificity for PAF detection. Studies using ICM have suggested a detection rate of approximately 25 % [14–16].

In our study we are focusing on a possible correlation between detection of atrial fibrillation by implantable ECG loop, and PITX2 and/or ZFHX3 gene mutations in cryptogenic stroke/TIA patients. A correlation could lead to implementation of this genomic approach to cryptogenic stroke/TIA diagnostics and management [17, 18].

## Methods

### Objective and endpoints

The objective of our study is to determine the proportion of patients with CS or cryptogenic TIA (CTIA) that have underlying PAF in comparison with control group. PAF is defined as an episode of irregular heart rhythm without detectable P waves lasting more than 30 s. Primary endpoints are the first documented PAF episode after CS or CTIA and incidence of PITX2 and ZFHX3 genes mutation. Secondary endpoints include recurrent stroke or TIA, new ischemic lesions found by brain imaging (CT and/or MRI), the size of left atrium (TTE/TEE), and relevant treatment changes (e.g. oral anticoagulant drugs). The follow-up of patient and control groups will last 12 months.

### Study participants

The study will analyse data from at least 100 patients with CS or CTIA admitted to the Department of Neurology in Nitra between 2014 and 2018 as well as at least 30 age- and sex-matched volunteers with no known vascular diesease in the control group. The study protocol has been approved by the Ethics Committee of the Faculty Hospital in Nitra (Slovak Republic). A written informed consent will be obtained from all study participants or their legal representatives.

### Inclusion criteria

1. CS or CTIA within 72 h from symptom onset
2. Age ≥ 18 years
3. CS: modified Rankin Scale (mRS) ≤ 4 at discharge (to ensure patient compliance)
4. CTIA: ABCD^2^ score ≥ 4
5. Ability and willingness of the patients or their legal representatives to understand the study instructions, both verbal and written, in accordance with ICH, GCP and legislation of the Slovak Republic

### Exclusion criteria

1. Known etiology of stroke/TIA
2. Untreated hyperthyroidism
3. Myocardial infarction within 1 month of stroke/TIA onset
4. Coronary bypass within 1 month of stroke/TIA onset
5. Valvular disease requiring urgent surgery
6. Documented AF or flutter
7. Patent foramen ovale
8. Permanent indication for oral anticoagulation therapy
9. Long-term steroid therapy > 30 days
10. Participation in another clinical trial aimed at experimental pharmacologic therapy
11. Chronic inflammatory disease (rheumatoid arthritis, inflammatory bowel diseases, lupus erythematosus etc.)
12. Severe co-morbidity not compatible with 12 months follow-up
13. Pregnant and breastfeeding women
14. Indication for implantation of pacemaker, implantable cardioverter defibrillator or implantable pulse generator

### Imaging and laboratory testing

Stroke diagnosis is accepted only when supported by consistent findings on CT and/or MRI. To identify the location and size of ischemic infarction, and to exclude intracranial hemorrhage or other pathology, standard non-contrast CT examination is performed initially after stroke symptom onset. When convincing lesions are not visible on CT, the patients undergo brain MRI (including DWI) to confirm ischemic etiology. Following extensive imaging and laboratory testing, we are using the TOAST criteria to classify the stroke as cryptogenic. Only TIA patients with symptoms of speech problems, limb weakness or hemianopia (ABCD^2^ score) are considered and enrolled when CTIA is confirmed. To detect PAF, patients undergo 48-h ECG Holter monitoring within 7 days after stroke/TIA onset and only those without documented AF have implanted the ICM device (REVEAL XT, REVEAL LINQ, Medtronic Inc., Minneapolis, USA). Both patient and control groups are monitored for 12 months. Patients’ follow-up visits are scheduled 1, 6, and 12 months after stroke/TIA onset and every 6 months thereafter until clinical study closure with extra visits in case of any new stroke/TIA symptoms. Disability of patients is classified according to the mRS and Barthel Index score. The healthy control participants undergo assessment at visit scheduled 1, 6, and 12 months after the implantation. Except standard blood laboratory analysis, complete vasculitis and thrombophilia testing panels are performed. Analysis of PITX2 (chromosome 4q25) and ZFHX3 (chromosome 16q22) gene polymorphisms are evaluated by PCR with genetic variation analysis such as restriction analysis (RFLP), high resolution melting (HRM), or allele-specific PCR. To analyse the data, descriptive statistics, statistical tests for differences, and correlation analyses will be used. This prospective and observational study is registered at www.clinicaltrials.gov with study identifier NCT02216370.

## Discussion

Unrecognized PAF and the consequent lack of prophylactic anticoagulant treatment may result in significant morbidity first presenting as a heart failure, stroke, systemic embolism, and occasionally death. It is generally accepted that intermittent ECG monitoring (Holter ECG) has low sensitivity to identify patients with PAF. The commonly used 24-h Holter ECG monitoring has been found to reach PAF diagnostic sensitivity of 2.4–9.4 % [19–21]. Longer continuous non-stop ECG monitoring has been shown to increase PAF detection. In a sub-study of TRENDS, a single 24-h Holter ECG diagnosed PAF in 3 %, whereas 7, 21, or 30 days of monitoring diagnosed PAF in 6, 9, or 11 %, respectively [22]. The EMBRACE study (The 30-Day Cardiac Event Monitor Belt for Recording Atrial Fibrillation After a Cerebral Ischemic Event) used noninvasive ambulatory ECG monitoring in patients with cryptogenic stroke or TIA for 30 days. Detection of atrial fibrillation improved fivefold and indication of anticoagulant treatment nearly doubled when compared with standard practice of short-duration ECG monitoring. AF was detected in 16.1 % of patients in the intervention group, compared with 3.2 % in the control group [23]. ICM devices for long-term ECG monitoring have been used primarily for differential diagnosis of syncope [24]. Cotter et al. reported “Reveal XT” PAF identification in 25.5 % (13 patients) of 51 patients with unexplained stroke [14]. Sensitivity and specificity of “Reveal XT” device for PAF was detected to be 96.1 and 85.4 % respectively (3-year non-stop monitoring) [25]. A large randomized clinical trial The Cryptogenic Stroke and Underlying Atrial Fibrillation trial (CRYSTAL AF) compared long-term ECG monitoring by ICM with conventional ECG Holter monitoring in patients with recent CS. After 12 months ICM monitoring detected over 7 times more patients with PAF than ECG Holter monitoring. Three-year follow-up detected 30 % PAF occurrence in the ICM group compared to 3 % in the Holter group [26]. Choe evaluated sensitivity and negative predictive value (NPV) of various external monitoring techniques in a cryptogenic stroke cohort. Simulated intermittent monitoring strategies were compared with continuous rhythm monitoring in 168 ICM patients of the CRYSTAL AF trial. Short-term monitoring included a single 24-h, 48-h and 7-day Holter, and 21-day and 30-day event recorders. Periodic monitoring consisted of monthly monitoring by 24-h Holters and quarterly monitoring by 24-h, 48-h and 7-day Holters. For a single monitoring period, sensitivity for AF diagnosis was lowest with 24-h Holters (1.3 %) and highest with 30-day event recorders (22.8 %). NPV ranged from 82.3 to 85.6 % for all single external monitoring strategies. Quarterly monitoring with 24-h Holters had 3.1 % sensitivity, whereas quarterly 7-day monitors increased it to 20.8 %. NPV of various types of repetitive periodic monitoring were similar at 82.6 to 85.3 %. Long-term continuous monitoring was superior in detecting AF compared to all types of intermittent monitoring evaluated (p <0.001) [27]. Ritter et al. presented 17.0 % PAF detection in ICM group in comparison with 1.7 % in 7-day ECG Holter group (p = 0.0077) [28].

Finding a new algorithm for detecting PAF in standard clinical practice should be of great interest as it would allow early indication of anticoagulant therapy in stroke/TIA patients. Additional information that would help to characterize stroke subtype could be provided by genetic data. Study of gene polymorphisms associated with an increased risk of AF suggests that subtle alterations in developmental factors, cell signalling, extracellular matrix regulation, and ion channels function are involved in arrhythmia pathogenesis. Large genome-wide association studies (GWAS) have found that the genetic markers close to the PITX2 gene on chromosome 4q25 and to the ZFHX3 gene on 16q22 are associated with both AF and cardio-embolic stroke [17, 18]. The PITX2 gene plays a critical role in development of left atrium and pulmonary vein myocardium. This particular region has been implicated to initiate and maintain AF. The expression patterns of the ZFHX3 gene in human cardiac and pulmonary tissue are not clear, nor are the mechanisms by which variants in this gene may predispose to AF.

### Conclusion

Cardioembolic TIA and stroke cause high rate disability and mortality worldwide. As cardio-embolic etiology is responsible for up to 58 % of CS, appropriate high-resolution cardiac monitoring plays a crucial role in arrhythmias detection after CTIA/CS. Early PAF diagnosis allows to start immediate anticoagulation therapy in order to reduce TIA/stroke recurrence. The expression profile of the selected genes may be very useful in selecting high-risk PAF individuals who could then undergo extensive ECG monitoring with no history of cerebrovascular disease.

## Abbreviations

ABCD^2^: Stroke risk scale after a transient ischemic attack
AF: Atrial fibrillation
BI: Barthel index
CS: Cryptogenic stroke
CTIA: Cryptogenic transient ischemic attack
CT: Computed tomography
DWI: Diffusion weighted imaging
ECG: Electrocardiography
GCP: Good clinical practice
GWAS: Genome-wide Association Studies
ICH: International Conference on Harmonisation
ICM: Insertable cardiac monitor
MRI: Magnetic resonance imaging
mRS: modified Rankin Scale
NIHSS: National Institutes of Health Stroke Scale
PAF: Paroxysmal atrial fibrillation
PITX2: Paired-like homeodomain 2
TIA: Transient ischemic attack
TOAST: Trial of Org 10172 in acute stroke treatment
ZFHX3: Zinc finger homeobox 3
